# Structural Insight into the Mechanism of *N*-Linked Glycosylation by Oligosaccharyltransferase

**DOI:** 10.3390/biom10040624

**Published:** 2020-04-17

**Authors:** Smita Mohanty, Bharat P Chaudhary, David Zoetewey

**Affiliations:** 1 Department of Chemistry, Oklahoma State University, Stillwater, OK 74078, USA; bharat.chaudhary@okstate.edu; 2 Department of Chemistry, Physics and Astronomy, Georgia College and State University, Milledgeville, GA 31061, USA; david.zoetewey@gcsu.edu

**Keywords:** mechanism of *N*-linked glycosylation, human oligosaccharyltransferase, yeast oligosaccharyltransferase, cryo-EM structures, membrane proteins, congenital disorders of glycosylation

## Abstract

Asparagine-linked glycosylation, also known as *N*-linked glycosylation is an essential and highly conserved post-translational protein modification that occurs in all three domains of life. This modification is essential for specific molecular recognition, protein folding, sorting in the endoplasmic reticulum, cell–cell communication, and stability. Defects in *N*-linked glycosylation results in a class of inherited diseases known as congenital disorders of glycosylation (CDG). *N*-linked glycosylation occurs in the endoplasmic reticulum (ER) lumen by a membrane associated enzyme complex called the oligosaccharyltransferase (OST). In the central step of this reaction, an oligosaccharide group is transferred from a lipid-linked dolichol pyrophosphate donor to the acceptor substrate, the side chain of a specific asparagine residue of a newly synthesized protein. The prokaryotic OST enzyme consists of a single polypeptide chain, also known as single subunit OST or ssOST. In contrast, the eukaryotic OST is a complex of multiple non-identical subunits. In this review, we will discuss the biochemical and structural characterization of the prokaryotic, yeast, and mammalian OST enzymes. This review explains the most recent high-resolution structures of OST determined thus far and the mechanistic implication of *N*-linked glycosylation throughout all domains of life. It has been shown that the ssOST enzyme, AglB protein of the archaeon *Archaeoglobus fulgidus*, and the PglB protein of the bacterium *Campylobactor lari* are structurally and functionally similar to the catalytic Stt3 subunit of the eukaryotic OST enzyme complex. Yeast OST enzyme complex contains a single Stt3 subunit, whereas the human OST complex is formed with either STT3A or STT3B, two paralogues of Stt3. Both human OST complexes, OST-A (with STT3A) and OST-B (containing STT3B), are involved in the *N*-linked glycosylation of proteins in the ER. The cryo-EM structures of both human OST-A and OST-B complexes were reported recently. An acceptor peptide and a donor substrate (dolichylphosphate) were observed to be bound to the OST-B complex whereas only dolichylphosphate was bound to the OST-A complex suggesting disparate affinities of two OST complexes for the acceptor substrates. However, we still lack an understanding of the independent role of each eukaryotic OST subunit in *N*-linked glycosylation or in the stabilization of the enzyme complex. Discerning the role of each subunit through structure and function studies will potentially reveal the mechanistic details of *N*-linked glycosylation in higher organisms. Thus, getting an insight into the requirement of multiple non-identical subunits in the *N*-linked glycosylation process in eukaryotes poses an important future goal.

## 1. Introduction

**Figure 1:**
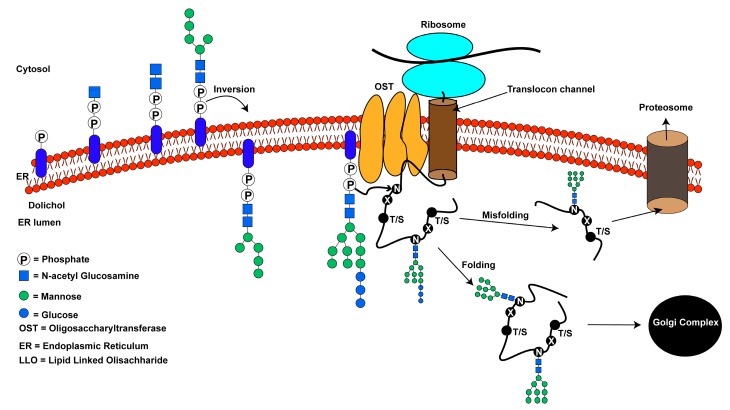
An overview of the *N*-linked glycosylation reaction of proteins in higher eukaryotes: pyrophosphate and monosaccharides are added to the dolichol lipid on the cytosolic side of the endoplasmic reticulum. The lipid linked oligosaccharide (LLO) is inverted to the luminal side of the endoplasmic reticulum (ER). Additional monosaccharides are added to form the mature LLO. Oligosaccharyltransferase (OST) catalyzes the transfer of the oligosaccharide from the LLO to the side-chain of an asparagine residue in -N-X-T/S- consensus sequence within a protein. Protein folding occurs after *N*-linked glycosylation. The three terminal glucose residues are trimmed before translocating to the Golgi apparatus for sorting. Misfolded proteins are targeted for degradation by proteasomes.

Oligosaccharyltransferase (OST) is a membrane-associated enzyme complex that catalyzes an essential and highly conserved asparagine-linked or *N*-linked glycosylation, a protein modification reaction. *N*-linked glycosylation occurs in the lumen of the endoplasmic reticulum (ER) and is ubiquitous in most eukaryotes [1,2,3] and some prokaryotes [4]. A general mechanism of *N*-linked glycosylation is schematically shown in Figure 1. Unlike eukaryotes, in prokaryotes such as archaea and eubacteria, *N*-linked glycosylation is largely nonessential although it aids in their survival and pathogenicity. The OST enzyme is monomeric in bacteria, archaea, and protozoa but is an oligomeric membrane-associated complex in animals, plants, and fungi [5]. OST catalyzes the transfer of a well-defined oligosaccharide donor substrate, consisting of three glucose (Glc), nine mannose (Man) and two *N*-acetylglucosamine (GlcNAc) monomers, (Glc_3_Man_9_GlcNAc_2_). This carbohydrate moiety is attached to the ER membrane through a dolichol pyrophosphate (DolPP), also known as a lipid-linked oligosaccharide (LLO). The oligosaccharide moiety of the LLO donor is transferred to the side chain of an asparagine residue defined by the sequence -N-X-T/S- (where X ≠ proline) of a newly synthesized polypeptide [6,7]. This modification is required for protein folding and other downstream processes including stability, molecular recognition, subcellular targeting, and cell–cell communication [5,8,9]. Defects in *N*-linked glycosylation cause a class of inherited diseases collectively known as congenital disorders of glycosylation (CDG) with clinical symptoms that include but are not limited to mental retardation, developmental delay, liver dysfunction, dysmorphic features, anorexia, and gastrointestinal disorders [10,11]. In the ER, *N*-linked glycans primarily assist in the proper folding of the nascent polypeptide [12,13,14]. Changes to the *N*-glycan structure on misfolded proteins flag them for proteasomal degradation by the ER quality control machinery [9].

The prokaryotic OST enzyme contains a membrane-embedded single subunit: archaeal glycosylation B (AglB) for archaea, and protein glycosylation B (PglB) for bacteria. In yeast, nine genes encoding OST subunits have been identified, cloned, and sequenced. The genes OST1, OST2, STT3, WBP1, and SWP1 are essential for the viability of the cell. Stt3 protein is the catalytic subunit in yeast OST, which is homologous to the single subunit OST enzyme, AglB in archaea, and PglB in bacteria [15]. The OST4 gene is essential above room temperature for the growth of yeast cells. Ost4 protein also binds to the catalytic subunit Stt3 and is necessary for the incorporation of either of the Ost3/Ost6 subunits [16]. The genes that encode Ost3, Ost5, and Ost6 are not essential, but are required for optimal enzyme activity [17,18,19]. Ost3 and Ost6 are homologous and only one of the two subunits is present in a given functional enzyme complex [16].

The metazoan OST subunits have also been identified and cloned. All of these protein subunits have homologs in the yeast OST complex [20] as shown in Table 1: ribophorin I is the homolog of yeast Ost1, DAD1 correspond to yeast Ost2, OST4 to Ost4, ribophorin II to yeast Swp1, TUSC3/MAGT1 to yeast Ost3/Ost6, TMEM258 to yeast Ost5, OST48 to yeast Wbp1, and STT3A/STT3B to yeast Stt3 [21]. These protein subunits assemble together into a multimeric complex similar to the yeast OST enzyme complex [22].

Genetic and biochemical studies have provided information on the subunits and their assembly in the yeast OST complex, however, recent advances in atomic resolution structural techniques including nuclear magnetic resonance (NMR) spectroscopy, crystallography, and cryo-electron microscopy have shed light on the molecular structures of the individual subunits or the whole OST complex. Crystal structures of the luminal domain of Ost6 [23,24], NMR structures of Ost4 [25,26] and Stt3 [27], and low resolutions cryo-EM structures of mammalian and yeast OST complex [28,29] have contributed to the understanding of the OST enzyme complex and the overall mechanism of *N*-linked glycosylation reaction (Table 2). Recent high-resolution cryo-EM structures of the yeast OST complex [30,31] and both the human OST complexes [32] have transformed our understanding of this enzyme (Table 2). These new insights have laid the groundwork for future investigation and mechanistic studies on the role of each individual subunit in acceptor/donor substrate recognition/specificity and/or stabilization of the multi-subunit enzyme complex in the *N*-linked glycosylation process. Here, we review the biochemical and structural characterization of bacterial, archaea, yeast, and mammalian OST complexes. In addition, we shed light on the *N*-linked glycosylation mechanism in all domains of life based on the most recent high-resolution structures.

## 2. *N*-Linked Glycosylation: An Overview

### 2.1. Donor Substrates in Prokaryotes and Archaea

*N*-linked glycosylation was originally believed to take place only in eukaryotic organisms until the discovery of alkali-sensitive glycoproteins extracted from the cell surface in archaea, *Halobacterium* [41,42,43,44,45]. In *Halobacterium salinarum*, the asparagine residue in the -N-X-T/S- motif is glycosylated with a tetra-saccharide that is transferred either from a membrane associated dolichol phosphate (DolP) or dolichol pyrophosphate (DolPP) donor depending on species [45,46,47]. The flagellins in the archaeon, *Methanococcus voltae*, are glycosylated at multiple -N-X-T/S- sites with a trisaccharide through an asparaginyl-β-GlcNAc linkage [44], but in *Haloferax volcanii*, dolichol-phosphate linked mannosyl-β(1,4)-galactosyl (Man-β(1,4)-Gal) oligosaccharide is the donor. Although the above oligosaccharides are shorter and not branched, the *N*-linked glycan of *Pyrobaculum calidifontis* has high mannose content with branching [48] while that of *Archaeoglobus fulgidus* has high hexose content with branching [49]. This evidence suggests a wide diversity in the *N*-glycan structures of the LLO donor substrates for the AglB enzyme although dolichol is the common carrier [42,44,49]. In contrast to archaea, in bacteria, the isoprenoid moiety is an undecaprenol (Und). The first eubacterial *N*-linked glycosylation system was identified for *Campylobacter jejuni* [4,43,50]. The PglB enzyme in eubacteria transfers the donor substrate, a preassembled heptasaccharide attached to undecaprenyl pyrophosphate (UndPP-heptasaccharide) to a wide array of target proteins at selected asparagine residues in the consensus sequon.

### 2.2. Donor Substrate in Eukaryotes and Possible Mechanism of Sugar Transfer

**Figure 2:**
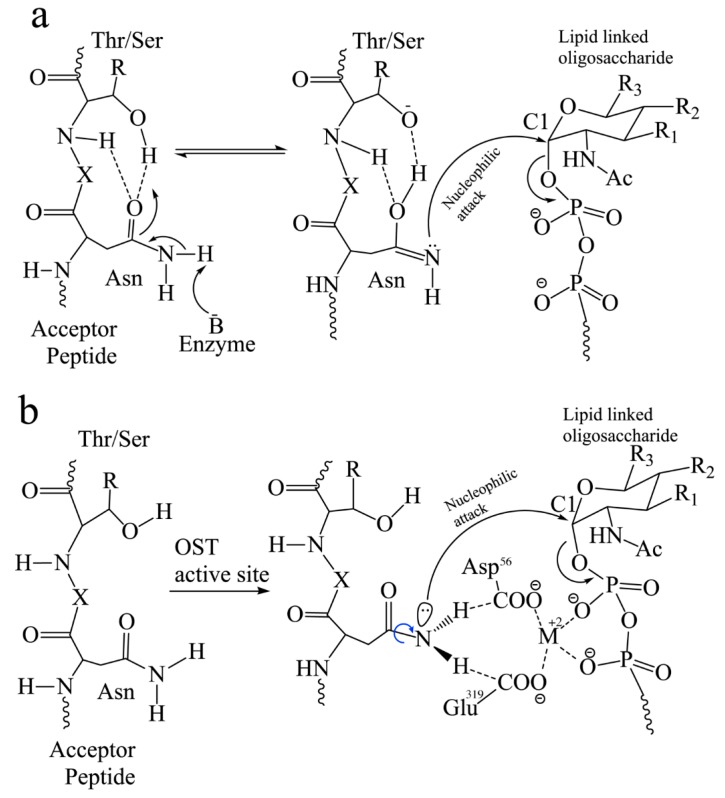
Possible reaction schemes of *N*-linked glycosylation showing nucleophilic attack by sidechain amide of the acceptor asparagine residue yielding a glycosylated peptide. (**a**) Mechanism of formation of an imidate tautomer, a competent nucleophile followed by nucleophilic attack on C1 of the dolichol-linked oligosaccharide. (**b**) Twisted amide activation mechanism for glycosylation of the acceptor peptide. The amide group forms H- bonds (dashed lines) with Glu319 and Asp56 residues leading to rotation of the C-N bond (indicated by the blue arrow) in bacterial PglB. These residues (Asp56 and Glu319) form H-bonds with the catalytic divalent metal ion. R_1_ is OH in eukaryotes, and oligosaccharyl in bacteria. R_2_ is oligosaccharyl in eukaryotes and NHAc in bacteria. R_3_ is CH_2_OH in eukaryotes and CH_3_ in bacteria [58]. X is any amino acid except proline.

In higher eukaryotes, three glucose (Glc), nine mannose (Man), and two *N*-acetyl glucosamine (GlcNAc) monosaccharides are assembled sequentially to form a 14-residue oligosaccharide structure on a lipid-linked dolichol pyrophosphate carrier constituting the lipid-linked oligosaccharide (LLO) donor substrate [51,52]. Lower eukaryotes such as protists use a shorter oligosaccharide lacking the terminal three glucose residues [53]. OST is responsible for the *en bloc* transfer of this preassembled 14-residue oligosaccharide chain (Glc_3_Man_9_GlcNAc_2_) to the selected asparagine specified by the *N*-linked glycosylation sequon on a nascent polypeptide. In the *N*-linked glycosylation reaction, the dolichol pyrophosphate (DolPP) acts as a leaving group following a nucleophilic attack at the C1 position of the GlcNAc. The mechanistic model to explain the primary amide reactivity in this OST catalyzed glycosyl transfer process was originally proposed as shown in Figure 2a [54,55,56].

In this model, the carboxamido oxygen of the asparagine side chain forms hydrogen bonds with the side-chain hydroxyl group and to the backbone amide proton of Ser/Thr at the +2 position. Subsequently, a proton from the nitrogen atom of the asparagine side chain is abstracted by a general base at the OST active site resulting in the formation of the imidate tautomer, a competent nucleophile. A nucleophilic attack on the DolPP-bound sugar was proposed to be the catalytic mechanism of the transfer of the glycan structure in this model (Figure 2a). However, this model (Figure 2a) has not been supported by the recent high-resolution structure of PglB-acceptor peptide complex [33]. No appropriate residues have been observed in the catalytic site of this complex that can activate the acceptor asparagine residue by abstracting a proton. Thus, this model involving a general base mechanism has not been supported by recent structural data. A new mechanism involving carboxamide twisting for the activation of the amide nitrogen has been proposed (Figure 2b). In this model, two residues at the catalytic site, D56 and E319 are optimally positioned to form hydrogen bonds with the two amide protons of the acceptor asparagine in PglB [33]. In AglB and yeast Stt3, D47 and E360 [30,31,39], human Stt3A, D49 and E351, and human Stt3B, D103 and N623 [32] are the two hydrogen bond acceptors for the acceptor asparagine amide protons. It has been proposed that formation of these hydrogen bonds would lead to the rotation of the N-C bond of the amido group, which effectively decouples the conjugation of the nitrogen electrons from the carbonyl group. This decoupling renders the nitrogen atom reactive for a nucleophilic attack on the C1 anomeric carbon of the LLO creating a new *N*-glycosidic bond while displacing the leaving group (DolPP) from the donor substrate [33,54,57]. During the reaction, the -N-X-T/S- sequon needs to maintain an extended strand conformation and does not accommodate all possible secondary structures of fully folded proteins suggesting that glycosylation needs to take place on an unfolded polypeptide acceptor substrate.

## 3. Structural Mechanisms of *N*-Linked Glycosylation by OST

### 3.1. Bacteria and Archaea

**Figure 3:**
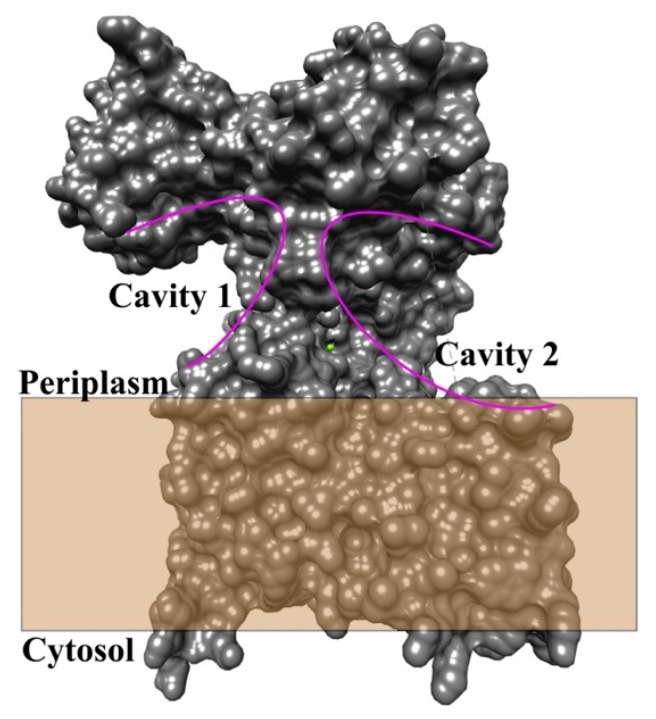
Surface representation of the bacterial PglB protein displays two cavities right above the membrane. The cavities are highlighted by purple solid arcs. The figure was prepared with chimera software and PDB file 3RCE.

The crystal structures of the eubacterial OST *Compylobactor lari,* PglB, in the presence of acceptor peptides (D/EXNXT/S) have greatly enhanced the understanding of the mechanism of *N*-linked glycosylation [33,35]. Furthermore, a crystal structure of the apo form of AglB from the archaeon *Archaeoglobus fulgidus* has shown that despite low sequence similarity, archaea use a structurally and likely functionally similar mechanism for oligosaccharide transfer [39]. These structures contain two domains: a transmembrane domain consisting of 13 transmembrane helices connected by short cytoplasmic loops and a luminal or periplasmic C-terminal domain with mixed α/β topology in both eubacteria and archaea [59]. The periplasmic C-terminal domain contains two distinct cavities (labeled cavity 1 and cavity 2 in Figure 3) that appear to be the binding sites for the nascent peptide and the oligosaccharide portion of the LLO, respectively, with the catalytic site situated between the two cavities.

**Figure 4:**
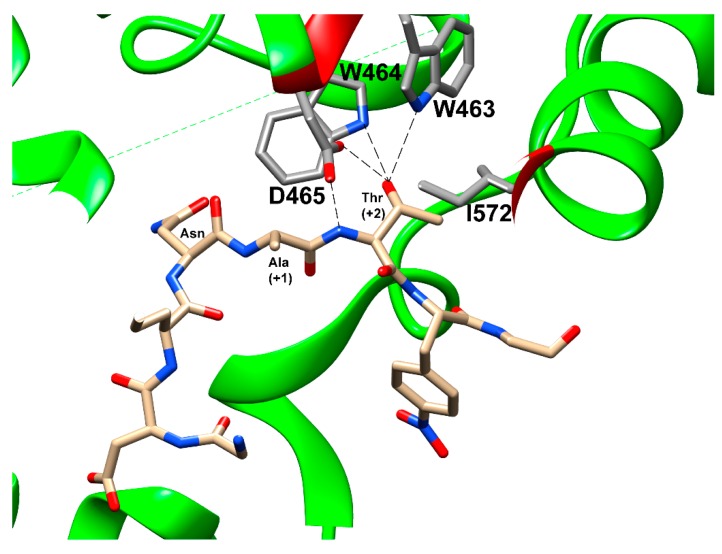
Residues interacting with the +2 Thr of bound peptide are shown and labelled. Hydrogen bonds from the WWD motif to the β-hydroxyl group are indicated by dashed lines. The figure was prepared using chimera and PDB ID 3RCE [33].

The acceptor substrate-binding pocket contains two conserved motifs: the highly conserved WWD motif and the bacterially specific MXXI motif. The indole nitrogen of the tryptophan and the aspartate side chain from the WWD motif in PglB form a set of highly specific hydrogen bonds with the hydroxyl (-OH) of either a T or S residue at the +2 position of the acceptor peptide sequence and its backbone amide (Figure 4). Therefore, the WWD motif specifically recognizes the side-chain hydroxyl group of T/S at the +2 position. This eliminates the possibility of the T/S side-chain performing any catalytic role in the mechanism of *N*-linked glycosylation as previously proposed [60] prior to structural determination. An isoleucine from the MXXI motif in PglB makes a hydrophobic contact with the methyl group of threonine at the +2 position of the peptide. This explains the observation that peptides containing serine at the +2 position have lower glycosylation efficiency relative to threonine [61,62,63]. However, this isoleucine is not conserved in AglB [39] and is occupied by lysine (K618) instead. This suggests that there is a degree of flexibility in the type of amino acid that may be present in close proximity to the methyl group of the threonine. Since non-canonical residues such as cysteine, alanine, and valine have low affinity when present in the recognition sequences, but larger residues such as leucine or glutamine have no affinity, this suggests that some rather bulky amino acid such as lysine is required in the position occupied by I572 in PglB [33,39]. However, the binding interactions with T/S at the +2 position by the WWD motif are only part of the peptide recognition by the enzyme. The amide nitrogen of the Asn residue at position 0 is situated between two acidic residues in PglB, D56, and E319. This facilitates hydrogen bond formation between the amide protons of the acceptor Asn with D56 and E319. These two amino acids (D56 and E319) along with R147, D154, and D156 also coordinate to a divalent metal ion. The metal ion is located between the two cavities and is positioned so that it can coordinate and stabilize the phosphate groups on the LLO. This stabilization provided by the metal ion and its surrounding chelating residues (D56 and E319) facilitates nucleophilic attack by the N residue via the carboxamide twist mechanism described earlier on the anomeric carbon of the saccharide directly attached to the phosphate or pyrophosphate leaving group. The second cavity provides a binding pocket to the saccharide portion of the LLO above the surface of the membrane and bridges the connection to the first cavity with the metal ion and its coordinating residues. Although there is no peptide bound to the active site in the crystal structure of AglB, it appears that the catalytically important residues E319 and D56 from PglB are replaced by E360 and D47 in AglB and the additional residues that coordinate to the metal ion including D154, D156, and R147 in PglB are replaced by D161, H163, and R154 in AglB, respectively. Therefore it appears that the overall mechanism is that the peptide recognition sequence is identified by specific binding of the WWD motif to the T/S at position +2 and the N at position 0 is sandwiched between two acidic residues that also chelate to a divalent metal ion and act as hydrogen bond acceptors to the two amide hydrogens of the N residue to activate the nitrogen as a nucleophile via the carboxamide twist that decouples the lone pair on the nitrogen atom from the carbonyl. The metal ion also coordinates and positions the phosphate groups from the LLO to be the leaving group once the now activated nucleophilic amide nitrogen attacks the anomeric carbon on the saccharide of the LLO to create the new *N*-glycosidic bond.

### 3.2. Yeast

In eukaryotes, OST is an enzyme complex composed of multiple non-identical protein subunits [6]. Yeast, *Saccharomyces cerevisiae*, has two functional OST isoforms each containing eight of the nine non-identical protein subunits. Both isoforms share seven subunits: Ost1, Ost2, Ost4, Ost5, Stt3, Swp1, and Wbp1. Each isoform contains either Ost3 or the homologous Ost6 subunit. Genetic, biochemical, and recent structural studies have verified that these subunits are grouped into three subcomplexes: subcomplex I (Ost1-Ost5), subcomplex II (Ost4-Stt3-Ost3/Ost6), and subcomplex III (Wbp1-Swp1-Ost2) [17,18,30,31,64,65,66,67]. The low resolution cryo-EM structure of yeast OST complex in the apo state provided an idea of the overall shape and approximate locations of four essential subunits [28]. However, recent advancements in cryo-EM technology have allowed construction of a high-resolution structure of the yeast OST complex in the apo state. The structural details have greatly contributed to the understanding of the yeast OST structure, including the assembly of the eight-subunit complex and the possible functions of certain subunits in *N*-linked glycosylation.

In this structure, the catalytic subunit Stt3 is at the core with seven other subunits assembled around it [30,31]. The structure contains a total of 28 transmembrane helices (TMHs) and five soluble luminal domains. Of these, TMH1 and the luminal domain of Ost3 are missing in the EM map along with the external loop EL5 (connecting TMH9 and TMH10) of Stt3. Poor resolution is also observed for the TMH9 of Stt3. These regions, EL5 and TMH9 of Stt3 and TMH1 of Ost3 are likely disordered in the absence of the bound substrates. This fact is supported by the substrate-bound structures of PglB and recently reported human Stt3B [32,33].

#### 3.2.1. Catalytic Subunit Stt3

**Figure 5:**
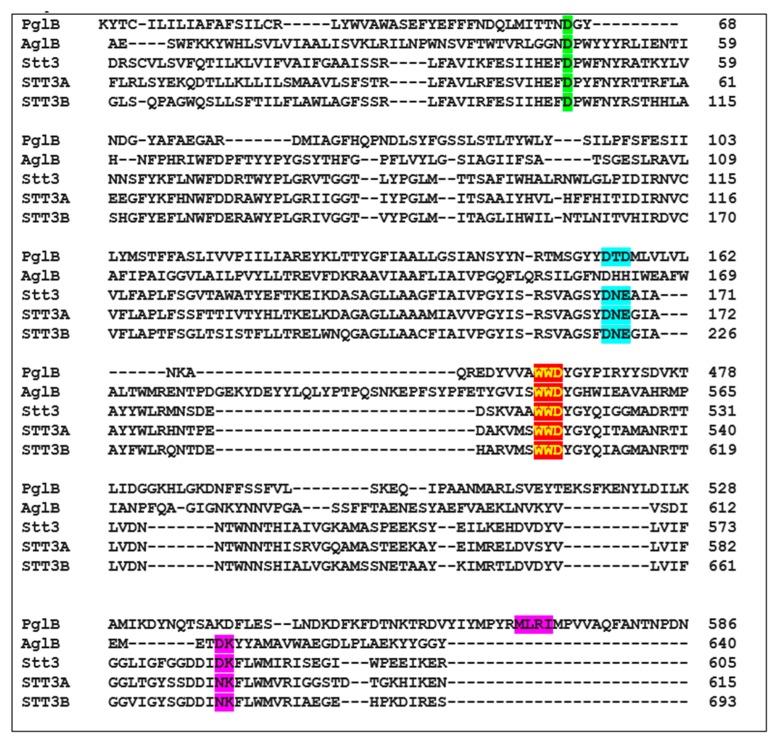
Sequence alignment of bacterial PglB, archaeon AglB, yeast Stt3, human STT3A, and human STT3B proteins to show the important residues and motifs. D56 (PglB), D47 (AglB and yeast Stt3), D49 (human STT3A), and D103 (human STT3B) are shown in green background. DXD motif in PglB, DXE motifs in yeast Stt3, human STT3A, and human STT3B are shown in cyan background. The conserved WWD motif is shown in red background highlighted in yellow. The MXXI motif in PglB that corresponds to DK motifs in AglB and yeast Stt3 are shown in purple background.

**Figure 6:**
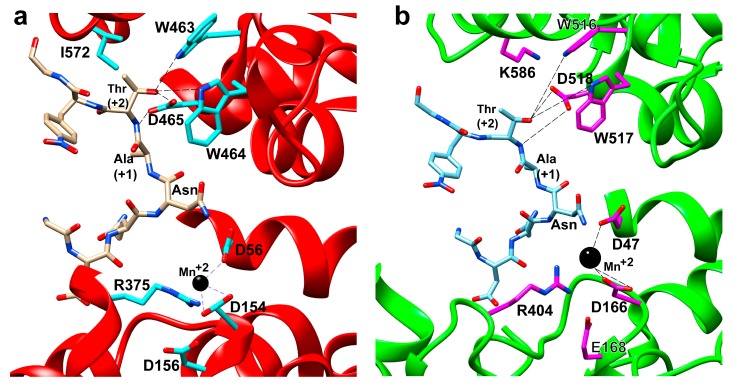
Active site of (**a**) bacterial PglB (PDB ID: 3RCE) and (**b**) yeast Stt3 (PDB ID: 6EZN), indicating the important residues involved in acceptor peptide recognition for glycosylation and metal ion co-ordination. Residues to metal co-ordination and H- bond of the WWD motif to +2 Thr of the acceptor peptide are shown with dotted lines.

Similar to the PglB and AglB structures, the catalytic subunit Stt3 is composed of 13 TMHs containing an *N*-terminal domain and a C-terminal luminal domain consisting of a mixed α/β fold. Ost4 is nestled between TMH1 and TMH13 of Stt3 stabilizing the Stt3 structure. Three TMHs of Ost3 interact with TMH10, 11, and 13 of Stt3. By comparing the yeast OST structure in the apo state to the substrate-bound structures of PglB and AglB, key mechanistic insights with respect to the binding pocket and interactions with both acceptor and donor substrates were gleaned. The superposition of the structures of yeast Stt3 and bacterial PglB revealed that the conserved motifs critical for the binding of the substrates have similar spatial arrangements suggesting a conserved mechanism of glycosylation despite low sequence identity. The WWD motif that forms hydrogen bonds with the β-hydroxyl group of the Ser/Thr at position +2 of the acceptor sequon, D47 (corresponding to D56 in PglB) that interacts with both the carboxamide group of the acceptor Asn and the catalytic metal ion, the D-X-D/E motif that coordinates the catalytic metal ion (where D166 and E168 in yeast correspond to D154 and D156 in PglB, respectively), and K586 residue of the DK motif (corresponding to I572 in the MXXI motif in PglB) that contributes additional binding interactions to the Ser/Thr at +2 position of the acceptor sequon are all conserved across the three domains of life (Figure 5). Indeed, residues that interact with LLO binding, R404 (R375 in PglB) with the pyrophosphate group of LLO, and Y521 (Y468 in PglB) that forms hydrogen bond with the *N*-acetyl group of the C-2 substituent of the first saccharide moiety, are also conserved (Figure 6a,b).

All the above observations indicate that yeast Stt3 and bacterial PglB share the same catalytic mechanism of glycosylation sequon recognition and LLO binding. However, there are also subtle differences in the Stt3 recognition of the acceptor sequon, which may translate to the requirement of consensus sequence and specificities. For example, the requirement of bacterial acceptor sequon, -D-X-N-X-T/S-, is longer than that of the eukaryal sequon, -N-X-T/S- [68]. This difference in the length of the required sequon is explained based on residues in PglB that interact with D at the −2 position. Thus, the peptide-binding pocket of the PglB has R331 which interacts with a negatively charged D/E at the −2 position of the bacterial sequon [33]. This R331 is conserved in bacterial ssOST. However, the putative acceptor peptide-binding pocket in yeast Stt3 contains D362 instead of R331. This suggests that for eukaryotes, a D/E at position −2 of the acceptor sequon is not necessary for the recognition by Stt3 and accordingly no positively charged residue is present in the peptide-binding pocket. Instead, a smaller amino acid such as D provides a larger cavity space to accommodate voluminous sidechains such as aromatic residues at the −2 position. This observation clarifies an earlier finding that OST can glycosylate substrates with aromatic residues at the −2 position with higher efficiency [69]. Another difference between bacterial PglB and yeast Stt3 is that the bacterial PglB MXXI motif is replaced by the DK motif in yeast Stt3 [30,31].

#### 3.2.2. Non-Catalytic Subunits

Despite a number of reports on the role of non-catalytic subunits of the yeast OST enzyme, the exact functions of these subunits are still not clear. Recent high-resolution structures of yeast OST have shed some light on the possible roles of some of these subunits [30,31].

Subcomplex I is composed of two subunits, Ost1 and Ost5. Ost1 contains two similar *N*-terminal luminal domains, which are formed of mainly β-sheets [30,31]. Ost1 is shown to bind only glycosylated peptides, which suggests that it may restrain the sliding back of the newly glycosylated peptide into the catalytic site [30,70]. Ost5 of this subcomplex has been suggested to assist Ost1 [30]. Both TMHs of Ost5 pack against the single TMH of Ost1 as seen in the recent cryo-EM structures [30,31].

Subcomplex II contains Ost4, the catalytic subunit Stt3, and either Ost3 or Ost6. Ost4, the smallest subunit of the OST complex, interacts very tightly with Stt3. The NMR structure of yeast Ost4 in mixed aqueous-organic solvent shows a well-formed kinked helix [26]. Mutation of any residue present in positions 18 to 24 to a charged residue in Ost4 resulted in severe growth defects in yeast [71]. These mutations were reported to destabilize the Stt3-Ost4-Ost3 sub-complex [71,72]. Ost4 stabilizes Stt3 and helps in the recruitment of Ost3/Ost6 as well [16,30,31]. Recombinant Ost4 and Ost4V23D mutant proteins have been successfully expressed, purified, and reconstituted in detergent for structure-function studies [73,74]. Ost3, a subunit that is homologous to Ost6, contains four transmembrane helices. Three of the TMHs of Ost3 interacts with TMH10, 11, and 13 of Stt3. The luminal domain of Ost3 is reported to be flexible in the absence of an LLO [31]. The transmembrane helix2 of this subunit is reported to interact with transmembrane helices 6 and 11 of Stt3 forming a groove that creates the putative LLO docking site [30].

Subcomplex III is composed of Ost2, Swp1, and Wbp1. Swp1 and Wbp1 are the non-catalytic subunits that contain large luminal *N*-terminal domains. In the membrane, four TMHs of Ost2 and three TMHs of Swp1 arrange around the only TMH of Wbp1 [30]. Ost2 contains an *N*-terminal α-helix located on the cytoplasmic side parallel to the membrane axis. This helix contacts TMHs 8 and 9 of Stt3. Thus, Ost2 mediates contacts between Stt3 and TMHs of Wbp1 and Swp1. While Wbp1 contains two luminal domains, Swp1 contains one. Despite several reports on functions of these proteins [75,76], their role in substrate binding and catalysis is still unclear [77]. Previously, Swp1 and Wbp1 along with Ost1 were predicted to act as chaperones assisting protein folding and glycosylation [75,78]. However, structures of the OST complex clearly show that they do not adopt chaperon-like folds [30,31]. Wbp1 possesses a GIFT domain [79]. The GIFT domain is named for flavobacterial gliding protein GldG and the intraflagellar transport (**IFT**) protein, IFT-52 of the green alga *Clamydomonas reinhartdtii* [79]. GIFT domains are proposed to have sugar-binding function based on their sequence similarity to β-galactosidase and sugar isomerase (SIS) [79]. Thus, it may play an important role in LLO binding. In fact, it has been proposed that Swp1 and Wbp1are likely involved in recruiting LLO or serve as a docking platform for the recruitment of other accessory proteins acting on nascent glycoproteins [30,31].

#### 3.2.3. Pathway for LLO Entry in Yeast OST

The structures of free PglB and substrate-bound PglB have been crucial to the understanding of the LLO pathway. The external loop5 (EL5) in PglB is disordered in the absence of the donor and acceptor substrates, but becomes ordered in the bound state. Based on this observation, it is proposed that EL5 disordering in PglB allows the donor substrate, LLO, to diffuse under it to the catalytic site [30,31]. The yeast OST also has a disordered EL5 in the apo state. However, a large membrane-embedded pocket, formed by TMH2 of Ost3, and TMHs 6, 8, and 11 of Stt3, is observed inside the OST [30]. Furthermore, the disordered EL5, TMH9 of Stt3, and TMH1 of Ost3 enlarge this donor-binding pocket. Yeast LLO is much larger in comparison to the bacterial LLO as far as both the lipid carrier (dolichol) and the oligosaccharide (OS) moiety are concerned. Based on these observations, it is proposed that unlike the bacterial LLO, the yeast LLO is too large to dive under the disordered EL5; hence, it enters the catalytic site via the gap between TMH8 and TMH9 of Stt3 [30].

#### 3.2.4. OST-Translocon Interaction

It was previously reported that the two yeast OST isoforms containing either Ost3 or Ost6 interact with the Sec61 and Ssh1 translocon complexes, respectively [80,81]. A good fit was observed between mammalian and yeast OSTs when the recent model of yeast OST was docked to the cryo-electron tomogram of a mammalian ribosome-translocon-OST complex [30,31]. Further docking studies using the crystal structure of mammalian Sec61 revealed that Ost3 mediates the interaction with the translocon [30]. The TMHs 3-4 of Ost3 specifically pack tightly with TMH1 of Sec61α, TMH2 of Sec61β, and the only TMH of Sec61γ [30].

#### 3.2.5. Assembly of Subcomplexes in the OST Complex

Recent cryo-EM structures [30,31] of yeast OST confirmed the previously reported groups of three subcomplexes: subcomplex I (Ost5-Ost1), subcomplex II (Stt3-Ost4-Ost3/Ost6), and subcomplex III (Ost2-Swp1-Wbp1) [30,31,82]. Recent structures suggest that there are not many protein–protein interactions among the subcomplexes; hence, the interface among the three subcomplexes in the transmembrane region is loose [30]. However, seven highly ordered phospholipids that appear to stabilize the complex are identified in the recent cryo-EM structure at the interface of these three subcomplexes with the eighth phospholipid situated at the donor-binding site of Stt3 [30]. Three of the well-ordered phospholipids are observed at the interface between subcomplex II (Stt3-Ost4-Ost3/Ost6) and subcomplex I (Ost1-Ost5). These phospholipid head groups are in contact with some of the hydrophilic residues of Ost1, Stt3, and Ost5. Additionally, the hydrophobic tails interact with the hydrophobic residues in TMH1–TMH2 of Stt3 and TMH2 of Ost5 [30]. Two of the phospholipids are involved in the stabilization of the interface of subcomplex II (Stt3-Ost4-Ost3/Ost6) and subcomplex III (Ost2-Swp1-Wbp1) [30]. While the hydrophobic tails of these phospholipids interact with hydrophobic residues of TMH3 of Ost2, TMH2–TMH3 of Swp1, TMH5 and EL1 of Stt3, the phosphate head groups form either hydrogen bonds or salt-bridges to the side chains of some of the ionizable residues of Wbp1 [30]. Thus, it appears that well-ordered phospholipids play crucial roles in the assembly and stabilization of all the three subcomplexes to form the complete OST enzyme complex.

### 3.3. Human

**Figure 7:**
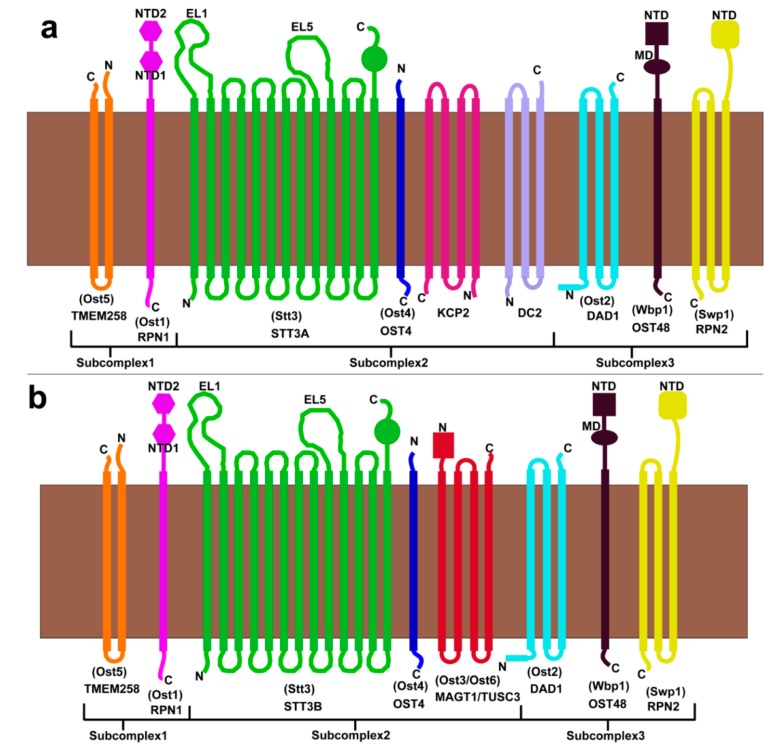
Subunit organization of the metazoan and yeast OST complex in ER membrane. (**a**) OST-A complex. (**b**) OST-B complex. Subunits are labeled by mammalian names with yeast subunit names shown in parentheses. Mammalian OST-A complex is homologous to the yeast OST complex, while the yeast OST lacks KCP2 and DC2 subunits found exclusively in the OST-B complex.

In humans and other mammals, the OST complex has diverged into two distinct isoforms known as OST-A and OST-B that perform distinctly different roles in *N*-linked glycosylation of proteins. OST-A is connected directly to the translocation channel called Sec61 in the ER membrane and scans the newly synthesized unfolded polypeptide chain emerging from the ribosome for glycosylation sites [83,84]. Therefore, OST-A is responsible for the majority of *N*-linked glycosylation in mammals [85]. In contrast OST-B seems to act in a proofreading role to catch glycosylation sites that OST-A misses for partially folded proteins or proteins that contain disulfide bonds. Recently, a high resolution cryo-EM structure of both OST-A and OST-B was reported that contains a bound lipid substrate in both complexes and a native peptide fragment in just OST-B [32]. OST-A and OST-B are very similar but contain some important differences. Both complexes contain the following subunits: ribophorin 1 (RPN1), ribophorin 2 (RPN2), defender against cell death 1(DAD1), OST 48-kDa subunit (OST48), OST 4-kDa subunit (OST4), and transmembrane protein 258 (TMEM258) (Figure 7a,b) [22]. Where they differ is that the OST-A complex contains keratinocyte-associated protein 2 (KCP2) and/or DC2 in place of OST3/OST6 from yeast and the catalytic subunit is STT3A. KCP2 and DC2 were previously shown to be the subunits that mediate a connection to the ribosome via the translocation channel Sec61 [83,84]. However, the most recent high-resolution structure of purified OST-A did not appear to contain the KCP2 subunit as a part of subcomplex II, but did appear to partially bind an additional protein called malectin [32]. The OST-B complex also binds malectin, but with a higher affinity than OST-A. Malectin, in association with ribophorin I, preferentially associates with the misfolded glycoproteins and guides these to the proteasome for degradation [86,87]. Thus, malectin is involved in the quality control of glycoproteins in the ER [87]. Since unfolded glycoproteins increase the interaction between malectin and ribophorin I [88], this suggests that the OST-B complex may encounter more unfolded glycoproteins than OST-A.

The recent cryo-EM structure indicates that DC2 binds specifically to STT3A and magnesium transporter protein 1 (MAGT1) binds specifically to STT3B [32]. The binding interactions between DC2 to STT3A and MAGT1 to STT3B are specific to each complex [32]. Neither could MAGT1 bind to STT3A nor could DC2 bind to STT3B due to steric clashes [32]. MAGT1 is a membrane protein that is expressed in a wide range of cells throughout the cell. It regulates the cellular magnesium levels [89]. STT3B is the catalytic subunit of OST-B and contains either tumor suppressor candidate 3 (TUSC3) or MAGT1 subunits as redox capable yeast OST3/OST6 homologs, respectively [20,84,90]. As a result, yeast subcomplexes I and III are identical in both OST-A and OST-B and the differences manifest in subcomplex II. Figure 7a,b shows the subunit composition and domain organization of the OST-A and OST-B complexes and their respective subcomplexes.

Overall, the architecture of both OST-A and OST-B are very similar in their cryo-EM structures especially with respect to the active site [32]. In the cryo-EM structure, surprisingly OST-A and OST-B complexes were each found to contain a dolichol phosphate (DolP) positioned near the catalytic divalent metal ion. No density was observed in the cryo-EM map for the second phosphate group of the expected dolichol pyrophosphate (DolPP) carrier for either complex [32]. A notable difference between OST-A and OST-B was that while OST-B was bound to a native peptide as its acceptor substrate at the active site, OST-A did not contain any acceptor substrate in its active site [32]. This suggests that OST-A, when not bound to the Sec61 translocation channel and the ribosome, has a lower inherent affinity for acceptor substrate peptide than OST-B. The lower affinity for an acceptor substrate peptide was further corroborated by in vitro assays with a cognate peptide and a minimal (GlcNAc_2_) LLO, which showed that only OST-B could form a glycosylated peptide within 1 h [32]. Using a larger (GlcNAc_2_Man_5_) LLO in the same assay demonstrated that both complexes are active, but OST-B is more active than OST-A [32].

**Figure 8:**
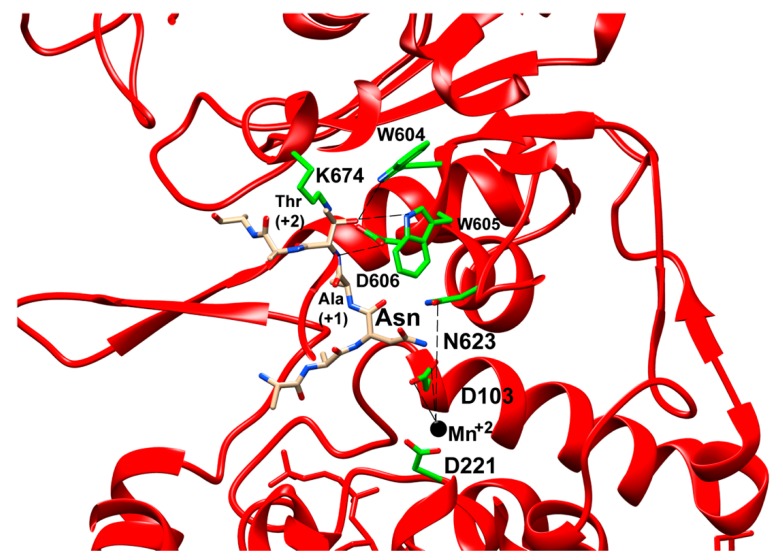
Close-up view of structure of STT3B (PDB ID: 6S7T) in cartoon representation. Residues interacting with Thr at +2 position of the acceptor peptide and with metal ion are shown as sticks and labeled. The H-bond formed by WWD motif to +2 Thr and metal to residue co-ordination are shown with dashed lines. D103 and N623 in STT3B correspond to D56 and E319 in bacterial PglB.

The bound peptide in the OST-B complex was in an extended strand conformation but looped into the shape of a U. As has been observed in PglB, the T residue at the +2 position is coordinated by the WWD loop with a series of hydrogen bonds (Figure 8). Additionally, the N residue at position 0 is situated near the divalent metal ion and between catalytic residues D103 and N623 (Figure 8), which are analogous to D56 and E319, respectively, from PglB, to be activated as a nucleophile by the same carboxamide twist mechanism observed in PglB and AglB. Finally, it is worth noting that the presence of the cognate peptide in the OST-B complex and the dolichol phosphate lipid in both of the purified complexes indicate that the rate-limiting step in the glycosylation pathway appears to be the exchange of the dolichol phosphate leaving group for a new LLO after the peptide has been properly positioned within the active site. Once a new LLO comes into the active site, the activated nucleophilic nitrogen of the asparagine attacks the anomeric carbon on the oligosaccharide and then dissociates as a glycosylated peptide, leaving the dolichol phosphate behind still coordinated to the divalent metal ion and making contact to an ordered EL5 helix that is disordered in the yeast OST when a lipid is not bound. This suggests that EL5 may transition from disordered to fully structured upon binding the LLO.

#### 3.3.1. Glycosylation by the OST-A Isoform

The STT3A complex interacts with the Sec61 translocon channel and is positioned adjacent to the protein translocon channel [90,91,92]. The acceptor sequences in nascent polypeptide emerging from the ribosome first makes contact with STT3A [15,93] so that the acceptor site in the newly translated polypeptide is scanned in an *N*-terminal to C-terminal manner [93,94]. This initial contact of the newly formed peptide does not appear to take place with the STT3B complex, which suggests a different form of peptide recognition not linked directly to the ribosome. Nascent polypeptides are co-translationally glycosylated by the STT3A complex more efficiently if Thr is present at the +2 position of the recognition sequence. Polypeptides containing Ser at the +2 position are skipped and instead are post-translationally glycosylated by the STT3B isoform [94]. The acceptor residue is exposed to the active site through a porthole in the catalytic site in a manner similar to what has been shown in the bacterial OST PglB [33]. The STT3A complex residing adjacent to the ribosome translocon complex scans for an acceptor sequence -N-X-T-, and transfers the oligosaccharide molecule co-translationally before disulfide bond formation can occur in the newly translated protein. *N*-linked glycosylation of proteins takes place prior to disulfide bond formation to allow the linear nascent polypeptide to enter the OST catalytic site [20]. Formation of disulfide bond/s ahead of *N*-linked glycosylation may allow the protein to adopt a conformation that could inhibit its entry into a OST catalytic site [20].

#### 3.3.2. Proofreading by the OST-B Complex

The STT3B complex glycosylates any conformationally available acceptor sites that are missed by STT3A [95,96]. This proofreading by the STT3B complex takes place either co-translationally or post-translationally depending upon the position of the skipped acceptor site relative to the C-terminus of the protein [90,96]. Acceptor sites located within the C-terminal 50 residues are rarely glycosylated by the STT3A complex. These skipped acceptor sites of polypeptides are instead post-translationally glycosylated by the STT3B complex. The acceptor sites that are frequently skipped by the STT3A complex are located within five residues of the signal sequence cleavage sites, in small membrane proteins, -N-X-G- sites, acceptor sites near to cystines, -N-C-T/S-, and closely spaced -N-X-S- sites [94,95,97,98,99]. The folding rate of nascent glycoproteins and the diffusion rate of the substrate after being skipped by STT3A are two of the factors that determine the efficiency of glycosylation by the STT3B complex [20].

## 4. Conclusions

The mechanism of *N*-linked glycosylation is remarkably similar in all three domains of life. Unicellular organisms such as bacteria contain a single unit OST enzyme, while the OST enzyme in both yeast and metazoans is composed of multiple subunits. The organisms containing multiple subunit OSTs are reported to glycosylate a variety of acceptor peptides [100]. This indicates that the non-catalytic subunits assist in increasing the glycosylation efficiency of Stt3 by interacting with the substrates or by impacting the protein folding after glycosylation. Recent high-resolution structures of the yeast OST complex, human OST complex, and the SEC61-STT3A complex have enhanced our understanding of the multi-subunit OST enzyme mechanism and possible roles for various subunits. However, the functions of certain subunits, particularly those having larger luminal domains, still require additional investigation.

## Figures and Tables

**Table 1:** OST subunits and their functions across three domains of life.

Archaea	Bacteria	Yeast	Human		Function
ssOST	ssOST	OST complex	OST-A complex	OST-B complex	
AglB	PglB	Stt3	STT3A	STT3B	Catalytic activity
		Ost4	OST4	OST4	Maintains stability of catalytic sub-complex
		Ost3/Ost6		MAGT1|N33/TUSC3	Oxidoreductase activity
		Ost5	TMEM258	TMEM258	Not clear
		Ost1	RPN1	RPN1	Restrains glycosylated peptide from sliding back to the catalytic site
		Ost2	DAD1	DAD1	Not clear
		Wbp1	OST48	OST48	Possibly LLO recruitment
		Swp1	RPN2	RPN2	Possibly LLO recruitment
			KCP2		Mediates interaction with translocon channel
			DC2		Mediates interaction with translocon channel

**Table 2:** List of all the high-resolution structures determined by various methods thus far with their PDB code.

Prokaryotic Oligosaccharyltransferase			References
Bacterial Oligosaccharyltransferase			
Protein	Method	PDB ID	
*Compylobactor lari* PglB with acceptor peptide	X-ray	3RCE	[33]
*Compylobactor lari* PglB with acceptor peptide and LLO analog	X-ray	5OGL	[34]
*Compylobactor lari* PglB with inhibitory peptide and reactive LLO	X-ray	6GXC	[35]
C-terminal domain of *Compylobactor jejuni* PglB	X-ray	3AAG	[36]
**Archaeal Oligosaccharyltransferase**			
C-terminal soluble domain from *Archaeoglobus fulgidus*	X-ray	3VU0	[37]
C-terminal soluble domain from *Pyrococcus horikoshii*	X-ray	3VU1	[37]
C-term globular domain as MBP fusion from *Archaeoglobus fulgidus*	X-ray	3WAI	[38]
*Archaeoglobus fulgidus* AglB	X-ray	3WAK	[39]
*Archaeoglobus fulgidus* AglB with Zn and sulfate	X-ray	3WAJ	[39]
*Archaeoglobus fulgidus* AglB with acceptor peptide	X-ray	5GMY	[40]
**Eukaryotic Oligosaccharyltransferase**			
(1) Yeast Oligosaccharyltransferase (OST)			
Yeast OST subunit Ost4p	Solution NMR	1RKL	[26]
Oxidized Ost6L	X-ray	3G7Y	[24]
Reduced Ost6L	X-ray	3G9B	[24]
Photo-reduced Ost6L	X-ray	3GA4	[24]
C-terminal domain of Stt3p subunit	Solution NMR	2LGZ	[27]
OST complex	Cryo-EM	6EZN	[31]
OST complex	Cryo-EM	6C26	[30]
(2) Human Oligosaccharyltransferase			
Soluble *N*-terminal domain of N33/Tusc3 subunit	X-ray	4M90, 4M91, 4M92, and 4M8G	[23]
Ost4 subunit	Solution NMR	2LAT	[25]
OST-A complex	Cryo-EM	6S7O	[32]
OST-B complex	Cryo-EM	6S7T	[32]

## References

[1] Dempski R.E., Imperiali B. (2002). Oligosaccharyl transferase: Gatekeeper to the secretory pathway. Curr. Opin. Chem. Biol..

[2] Kornfeld R., Kornfeld S. (1985). Assembly of asparagine-linked oligosaccharides. Annu. Rev. Biochem..

[3] Larkin A., Imperiali B. (2011). The expanding horizons of asparagine-linked glycosylation. Biochemistry.

[4] Wacker M., Linton D., Hitchen P.G., Nita-Lazar M., Haslam S.M., North S.J., Panico M., Morris H.R., Dell A., Wren B.W. (2002). N-linked glycosylation in Campylobacter jejuni and its functional transfer into E. coli. Science.

[5] Mohorko E., Glockshuber R., Aebi M. (2011). Oligosaccharyltransferase: The central enzyme of N-linked protein glycosylation. J. Inherit. Metab. Dis..

[6] Knauer R., Lehle L. (1999). The oligosaccharyltransferase complex from yeast. Biochim. Biophys. Acta.

[7] Welply J.K., Shenbagamurthi P., Lennarz W.J., Naider F. (1983). Substrate recognition by oligosaccharyltransferase. Studies on glycosylation of modified Asn-X-Thr/Ser tripeptides. J. Biol. Chem..

[8] Gahmberg C.G., Tolvanen M. (1996). Why mammalian cell surface proteins are glycoproteins. Trends Biochem. Sci..

[9] Helenius A., Aebi M. (2004). Roles of N-linked glycans in the endoplasmic reticulum. Annu. Rev. Biochem..

[10] Freeze H.H. (2013). Understanding human glycosylation disorders: Biochemistry leads the charge. J. Biol. Chem..

[11] Hennet T., Cabalzar J. (2015). Congenital disorders of glycosylation: A concise chart of glycocalyx dysfunction. Trends Biochem. Sci..

[12] Helenius A. (1994). How N-linked oligosaccharides affect glycoprotein folding in the endoplasmic reticulum. Mol. Biol. Cell.

[13] Paulson J.C. (1989). Glycoproteins: What are the sugar chains for?. Trends Biochem. Sci..

[14] Wormald M.R., Dwek R.A. (1999). Glycoproteins: Glycan presentation and protein-fold stability. Structure.

[15] Nilsson I., Kelleher D.J., Miao Y., Shao Y., Kreibich G., Gilmore R., von Heijne G., Johnson A.E. (2003). Photocross-linking of nascent chains to the STT3 subunit of the oligosaccharyltransferase complex. J. Cell Biol..

[16] Spirig U., Bodmer D., Wacker M., Burda P., Aebi M. (2005). The 3.4-kDa Ost4 protein is required for the assembly of two distinct oligosaccharyltransferase complexes in yeast. Glycobiology.

[17] Kelleher D.J., Gilmore R. (2006). An evolving view of the eukaryotic oligosaccharyltransferase. Glycobiology.

[18] Mueller S., Wahlander A., Selevsek N., Otto C., Ngwa E.M., Poljak K., Frey A.D., Aebi M., Gauss R. (2015). Protein degradation corrects for imbalanced subunit stoichiometry in OST complex assembly. Mol. Biol. Cell.

[19] Zufferey R., Knauer R., Burda P., Stagljar I., te Heesen S., Lehle L., Aebi M. (1995). STT3, a highly conserved protein required for yeast oligosaccharyl transferase activity in vivo. EMBO J..

[20] Cherepanova N., Shrimal S., Gilmore R. (2016). N-linked glycosylation and homeostasis of the endoplasmic reticulum. Curr. Opin. Cell Biol..

[21] Shrimal S., Cherepanova N.A., Gilmore R. (2015). Cotranslational and posttranslocational N-glycosylation of proteins in the endoplasmic reticulum. Semin. Cell Dev. Biol..

[22] Kelleher D.J., Karaoglu D., Mandon E.C., Gilmore R. (2003). Oligosaccharyltransferase isoforms that contain different catalytic STT3 subunits have distinct enzymatic properties. Mol. Cell.

[23] Mohorko E., Owen R.L., Malojcic G., Brozzo M.S., Aebi M., Glockshuber R. (2014). Structural basis of substrate specificity of human oligosaccharyl transferase subunit N33/Tusc3 and its role in regulating protein N-glycosylation. Structure.

[24] Schulz B.L., Stirnimann C.U., Grimshaw J.P., Brozzo M.S., Fritsch F., Mohorko E., Capitani G., Glockshuber R., Grutter M.G., Aebi M. (2009). Oxidoreductase activity of oligosaccharyltransferase subunits Ost3p and Ost6p defines site-specific glycosylation efficiency. Proc. Natl. Acad. Sci. USA.

[25] Gayen S., Kang C. (2011). Solution structure of a human minimembrane protein Ost4, a subunit of the oligosaccharyltransferase complex. Biochem. Biophys. Res. Commun..

[26] Zubkov S., Lennarz W.J., Mohanty S. (2004). Structural basis for the function of a minimembrane protein subunit of yeast oligosaccharyltransferase. Proc. Natl. Acad. Sci. USA.

[27] Huang C., Bhaskaran R., Mohanty S. (2012). Eukaryotic N-glycosylation occurs via the membrane-anchored C-terminal domain of the Stt3p subunit of oligosaccharyltransferase. J. Biol. Chem..

[28] Li H., Chavan M., Schindelin H., Lennarz W.J., Li H. (2008). Structure of the oligosaccharyl transferase complex at 12 A resolution. Structure.

[29] Pfeffer S., Dudek J., Gogala M., Schorr S., Linxweiler J., Lang S., Becker T., Beckmann R., Zimmermann R., Forster F. (2014). Structure of the mammalian oligosaccharyl-transferase complex in the native ER protein translocon. Nat. Commun..

[30] Bai L., Wang T., Zhao G., Kovach A., Li H. (2018). The atomic structure of a eukaryotic oligosaccharyltransferase complex. Nature.

[31] Wild R., Kowal J., Eyring J., Ngwa E.M., Aebi M., Locher K.P. (2018). Structure of the yeast oligosaccharyltransferase complex gives insight into eukaryotic N-glycosylation. Science.

[32] Ramirez A.S., Kowal J., Locher K.P. (2019). Cryo-electron microscopy structures of human oligosaccharyltransferase complexes OST-A and OST-B. Science.

[33] Lizak C., Gerber S., Numao S., Aebi M., Locher K.P. (2011). X-ray structure of a bacterial oligosaccharyltransferase. Nature.

[34] Napiórkowska M., Boilevin J., Sovdat T., Darbre T., Reymond J.-L., Aebi M., Locher K.P. (2017). Molecular basis of lipid-linked oligosaccharide recognition and processing by bacterial oligosaccharyltransferase. Nat. Struct. Mol. Biol..

[35] Napiórkowska M., Boilevin J., Darbre T., Reymond J.L., Locher K.P. (2018). Structure of bacterial oligosaccharyltransferase PglB bound to a reactive LLO and an inhibitory peptide. Sci. Rep..

[36] Maita N., Nyirenda J., Igura M., Kamishikiryo J., Kohda D. (2010). Comparative structural biology of eubacterial and archaeal oligosaccharyltransferases. J. Biol. Chem..

[37] Nyirenda J., Matsumoto S., Saitoh T., Maita N., Noda N.N., Inagaki F., Kohda D. (2013). Crystallographic and NMR evidence for flexibility in oligosaccharyltransferases and its catalytic significance. Structure.

[38] Matsumoto S., Shimada A., Kohda D. (2013). Crystal structure of the C-terminal globular domain of the third paralog of the Archaeoglobus fulgidus oligosaccharyltransferases. BMC Struct. Biol..

[39] Matsumoto S., Shimada A., Nyirenda J., Igura M., Kawano Y., Kohda D. (2013). Crystal structures of an archaeal oligosaccharyltransferase provide insights into the catalytic cycle of N-linked protein glycosylation. Proc. Natl. Acad. Sci. USA.

[40] Matsumoto S., Taguchi Y., Shimada A., Igura M., Kohda D. (2017). Tethering an N-glycosylation sequon-containing peptide creates a catalytically competent oligosaccharyltransferase complex. Biochemistry.

[41] Lechner J., Wieland F. (1989). Structure and biosynthesis of prokaryotic glycoproteins. Annu. Rev. Biochem..

[42] Kuntz C., Sonnenbichler J., Sonnenbichler I., Sumper M., Zeitler R. (1997). Isolation and characterization of dolichol-linked oligosaccharides from Haloferax volcanii. Glycobiology.

[43] Szymanski C.M., Yao R., Ewing C.P., Trust T.J., Guerry P. (1999). Evidence for a system of general protein glycosylation in Campylobacter jejuni. Mol. Microbiol..

[44] Voisin S., Houliston R.S., Kelly J., Brisson J.-R., Watson D., Bardy S.L., Jarrell K.F., Logan S.M. (2005). Identification and characterization of the unique N-linked glycan common to the flagellins and S-layer glycoprotein of Methanococcus voltae. J. Biol. Chem..

[45] Wieland F., Paul G., Sumper M. (1985). Halobacterial flagellins are sulfated glycoproteins. J. Biol. Chem..

[46] Chang M.M., Imperiali B., Eichler J., Guan Z. (2015). N-linked glycans are assembled on highly reduced dolichol phosphate carriers in the hyperthermophilic archaea pyrococcus furiosus. PLoS ONE.

[47] Taguchi Y., Fujinami D., Kohda D. (2016). Comparative analysis of archaeal lipid-linked oligosaccharides that serve as oligosaccharide donors for asn glycosylation. J. Biol. Chem..

[48] Fujinami D., Taguchi Y., Kohda D. (2017). Asn-linked oligosaccharide chain of a crenarchaeon, Pyrobaculum calidifontis, is reminiscent of the eukaryotic high-mannose-type glycan. Glycobiology.

[49] Fujinami D., Nyirenda J., Matsumoto S., Kohda D. (2015). Structural elucidation of an asparagine-linked oligosaccharide from the hyperthermophilic archaeon, Archaeoglobus fulgidus. Carbohydr. Res..

[50] Young N.M., Brisson J.R., Kelly J., Watson D.C., Tessier L., Lanthier P.H., Jarrell H.C., Cadotte N., St Michael F., Aberg E. (2002). Structure of the N-linked glycan present on multiple glycoproteins in the Gram-negative bacterium, Campylobacter jejuni. J. Biol. Chem..

[51] Chapman A., Li E., Kornfeld S. (1979). The biosynthesis of the major lipid-linked oligosaccharide of Chinese hamster ovary cells occurs by the ordered addition of mannose residues. J. Biol. Chem..

[52] Liu T., Stetson B., Turco S.J., Hubbard S.C., Robbins P.W. (1979). Arrangement of glucose residues in the lipid-linked oligosaccharide precursor of asparaginyl oligosaccharides. J. Biol. Chem..

[53] Samuelson J., Banerjee S., Magnelli P., Cui J., Kelleher D.J., Gilmore R., Robbins P.W. (2005). The diversity of dolichol-linked precursors to asn-linked glycans likely results from secondary loss of sets of glycosyltransferases. Proc. Natl. Acad. Sci. USA.

[54] Bause E., Legler G. (1981). The role of the hydroxy amino acid in the triplet sequence Asn-Xaa-Thr(Ser) for the N-glycosylation step during glycoprotein biosynthesis. Biochem. J..

[55] Imperiali B., Shannon K.L., Unno M., Rickert K.W. (1992). Mechanistic proposal for asparagine-linked glycosylation. J. Am. Chem. Soc..

[56] Marshall R.D. (1972). Glycoproteins. Annu. Rev. Biochem..

[57] Kohda D. (2018). Structural basis of protein asn-glycosylation by oligosaccharyltransferases. Adv. Exp. Med. Biol..

[58] Lizak C., Gerber S., Michaud G., Schubert M., Fan Y.-Y., Bucher M., Darbre T., Aebi M., Reymond J.-L., Locher K.P. (2013). Unexpected reactivity and mechanism of carboxamide activation in bacterial N-linked protein glycosylation. Nat. Commun..

[59] Igura M., Maita N., Kamishikiryo J., Yamada M., Obita T., Maenaka K., Kohda D. (2008). Structure-guided identification of a new catalytic motif of oligosaccharyltransferase. EMBO J..

[60] Imperiali B., Moats R.A., Fisher S.L., Prins T.J. (1992). A conformational study of peptides with the general structure Ac-L-Xaa-Pro-D-Xaa-L-Xaa-NH2: Spectroscopic evidence for a peptide with significant.beta.-turn character in water and in dimethyl sulfoxide. J. Am. Chem. Soc..

[61] Breuer W., Klein R.A., Hardt B., Bartoschek A., Bause E. (2001). Oligosaccharyltransferase is highly specific for the hydroxy amino acid in Asn-Xaa-Thr/Ser. FEBS Lett..

[62] Chen M.M., Glover K.J., Imperiali B. (2007). From peptide to protein: Comparative analysis of the substrate specificity of N-linked glycosylation in C. jejuni. Biochemistry.

[63] Gerber S., Lizak C., Michaud G., Bucher M., Darbre T., Aebi M., Reymond J.L., Locher K.P. (2013). Mechanism of bacterial oligosaccharyltransferase: In vitro quantification of sequon binding and catalysis. J. Biol. Chem..

[64] Karaoglu D., Kelleher D.J., Gilmore R. (1997). The highly conserved Stt3 protein is a subunit of the yeast oligosaccharyltransferase and forms a subcomplex with Ost3p and Ost4p. J. Biol. Chem..

[65] Spirig U., Glavas M., Bodmer D., Reiss G., Burda P., Lippuner V., te Heesen S., Aebi M. (1997). The STT3 protein is a component of the yeast oligosaccharyltransferase complex. Mol. Genet. Genom. MGG.

[66] Te Heesen S., Janetzky B., Lehle L., Aebi M. (1992). The yeast WBP1 is essential for oligosaccharyl transferase activity in vivo and in vitro. EMBO J..

[67] te Heesen S., Knauer R., Lehle L., Aebi M. (1993). Yeast Wbp1p and Swp1p form a protein complex essential for oligosaccharyl transferase activity. EMBO J..

[68] Kowarik M., Young N.M., Numao S., Schulz B.L., Hug I., Callewaert N., Mills D.C., Watson D.C., Hernandez M., Kelly J.F. (2006). Definition of the bacterial N-glycosylation site consensus sequence. EMBO J..

[69] Murray A.N., Chen W., Antonopoulos A., Hanson S.R., Wiseman R.L., Dell A., Haslam S.M., Powers D.L., Powers E.T., Kelly J.W. (2015). Enhanced aromatic sequons increase oligosaccharyltransferase glycosylation efficiency and glycan homogeneity. Chem. Biol..

[70] Yan Q., Prestwich G.D., Lennarz W.J. (1999). The Ost1p subunit of yeast oligosaccharyl transferase recognizes the peptide glycosylation site sequence, -Asn-X-Ser/Thr. J. Biol. Chem..

[71] Kim H., Park H., Montalvo L., Lennarz W.J. (2000). Studies on the role of the hydrophobic domain of Ost4p in interactions with other subunits of yeast oligosaccharyl transferase. Proc. Natl. Acad. Sci. USA.

[72] Kim H., Yan Q., Von Heijne G., Caputo G.A., Lennarz W.J. (2003). Determination of the membrane topology of Ost4p and its subunit interactions in the oligosaccharyltransferase complex in Saccharomyces cerevisiae. Proc. Natl. Acad. Sci. USA.

[73] Chaudhary B., Mazumder S., Mohanty S. (2017). Production and biophysical characterization of a mini-membrane protein, Ost4V23D: A functionally important mutant of yeast oligosaccharyltransferase subunit Ost4p. Protein Expr. Purif..

[74] Kumar A., Ward P., Katre U.V., Mohanty S. (2012). A novel and simple method of production and biophysical characterization of a mini-membrane protein, Ost4p: A subunit of yeast oligosaccharyl transferase. Biopolymers.

[75] Pathak R., Hendrickson T.L., Imperiali B. (1995). Sulfhydryl modification of the yeast Wbp1p inhibits oligosaccharyl transferase activity. Biochemistry.

[76] Yan Q., Lennarz W.J. (2002). Studies on the function of oligosaccharyl transferase subunits. Stt3p is directly involved in the glycosylation process. J. Biol. Chem..

[77] Shrimal S., Gilmore R. (2019). Oligosaccharyltransferase structures provide novel insight into the mechanism of asparagine-linked glycosylation in prokaryotic and eukaryotic cells. Glycobiology.

[78] Chavan M., Yan A., Lennarz W.J. (2005). Subunits of the translocon interact with components of the oligosaccharyl transferase complex. J. Biol. Chem..

[79] Beatson S., Ponting C.P. (2004). GIFT domains: Linking eukaryotic intraflagellar transport and glycosylation to bacterial gliding. Trends Biochem. Sci..

[80] Harada Y., Li H., Li H., Lennarz W.J. (2009). Oligosaccharyltransferase directly binds to ribosome at a location near the translocon-binding site. Proc. Natl. Acad. Sci. USA.

[81] Yan A., Lennarz W.J. (2005). Two oligosaccharyl transferase complexes exist in yeast and associate with two different translocons. Glycobiology.

[82] Yan A., Ahmed E., Yan Q., Lennarz W.J. (2003). New findings on interactions among the yeast oligosaccharyl transferase subunits using a chemical cross-linker. J. Biol. Chem..

[83] Braunger K., Pfeffer S., Shrimal S., Gilmore R., Berninghausen O., Mandon E.C., Becker T., Forster F., Beckmann R. (2018). Structural basis for coupling protein transport and N-glycosylation at the mammalian endoplasmic reticulum. Science.

[84] Shrimal S., Cherepanova N.A., Gilmore R. (2017). DC2 and KCP2 mediate the interaction between the oligosaccharyltransferase and the ER translocon. J. Cell Biol..

[85] Cherepanova N.A., Venev S.V., Leszyk J.D., Shaffer S.A., Gilmore R. (2019). Quantitative glycoproteomics reveals new classes of STT3A- and STT3B-dependent N-glycosylation sites. J. Cell Biol..

[86] Galli C., Bernasconi R., Soldà T., Calanca V., Molinari M. (2011). Malectin participates in a backup glycoprotein quality control pathway in the mammalian ER. PLoS ONE.

[87] Takeda K., Qin S.-Y., Matsumoto N., Yamamoto K. (2014). Association of malectin with ribophorin I is crucial for attenuation of misfolded glycoprotein secretion. Biochem. Biophys. Res. Commun..

[88] Qin S.-Y., Hu D., Matsumoto K., Takeda K., Matsumoto N., Yamaguchi Y., Yamamoto K. (2012). Malectin forms a complex with ribophorin I for enhanced association with misfolded glycoproteins. J. Biol. Chem..

[89] Goytain A., Quamme G.A. (2005). Identification and characterization of a novel mammalian Mg2+ transporter with channel-like properties. BMC Genom..

[90] Ruiz-Canada C., Kelleher D.J., Gilmore R. (2009). Cotranslational and posttranslational N-glycosylation of polypeptides by distinct mammalian OST isoforms. Cell.

[91] Shibatani T., David L.L., McCormack A.L., Frueh K., Skach W.R. (2005). Proteomic analysis of mammalian oligosaccharyltransferase reveals multiple subcomplexes that contain Sec61, TRAP, and two potential new subunits. Biochemistry.

[92] Conti B.J., Devaraneni P.K., Yang Z., David L.L., Skach W.R. (2015). Cotranslational stabilization of Sec62/63 within the ER Sec61 translocon is controlled by distinct substrate-driven translocation events. Mol. Cell.

[93] Shrimal S., Cherepanova N.A., Mandon E.C., Venev S.V., Gilmore R. (2019). Asparagine-linked glycosylation is not directly coupled to protein translocation across the endoplasmic reticulum in Saccharomyces cerevisiae. Mol. Biol. Cell.

[94] Shrimal S., Gilmore R. (2013). Glycosylation of closely spaced acceptor sites in human glycoproteins. J. Cell Sci..

[95] Cherepanova N.A., Shrimal S., Gilmore R. (2014). Oxidoreductase activity is necessary for N-glycosylation of cysteine-proximal acceptor sites in glycoproteins. J. Cell Biol..

[96] Shrimal S., Trueman S.F., Gilmore R. (2013). Extreme C-terminal sites are posttranslocationally glycosylated by the STT3B isoform of the OST. J. Cell Biol..

[97] Malaby H.L.H., Kobertz W.R. (2014). The middle X residue influences cotranslational N-glycosylation consensus site skipping. Biochemistry.

[98] Bas T., Gao G.Y., Lvov A., Chandrasekhar K.D., Gilmore R., Kobertz W.R. (2011). Post-translational N-glycosylation of type I transmembrane KCNE1 peptides: Implications for membrane protein biogenesis and disease. J. Biol. Chem..

[99] Malaby H.L.H., Kobertz W.R. (2013). Molecular determinants of co- and post-translational N-glycosylation of type I transmembrane peptides. Biochem. J..

[100] Dell A., Galadari A., Sastre F., Hitchen P. (2010). Similarities and differences in the glycosylation mechanisms in prokaryotes and eukaryotes. Int. J. Microbiol..

